# Investigating the Potential for Sulforaphane to Attenuate Gastrointestinal Dysfunction in *mdx* Dystrophic Mice

**DOI:** 10.3390/nu13124559

**Published:** 2021-12-20

**Authors:** Kristy Swiderski, Suzannah J. Read, Audrey S. Chan, Jin D. Chung, Jennifer Trieu, Timur Naim, René Koopman, Gordon S. Lynch

**Affiliations:** Centre for Muscle Research, Department of Anatomy and Physiology, The University of Melbourne, Parkville, VIC 3010, Australia; kristys@unimelb.edu.au (K.S.); u7089737@anu.edu.au (S.J.R.); audrey.chan@unimelb.edu.au (A.S.C.); chungjd@student.unimelb.edu.au (J.D.C.); jennifer.trieu@unimelb.edu.au (J.T.); timswampy66@gmail.com (T.N.); rkoopman@unimelb.edu.au (R.K.)

**Keywords:** sulforaphane, Duchenne muscular dystrophy, gastrointestinal dysfunction, colon, nutraceutical

## Abstract

Gastrointestinal (GI) dysfunction is an important, yet understudied condition associated with Duchenne muscular dystrophy (DMD), with patients reporting bloating, diarrhea, and general discomfort, contributing to a reduced quality of life. In the *mdx* mouse, the most commonly used mouse model of DMD, studies have confirmed GI dysfunction (reported as altered contractility and GI transit through the small and large intestine), associated with increased local and systemic inflammation. Sulforaphane (SFN) is a natural isothiocyanate with anti-inflammatory and anti-oxidative properties via its activation of Nrf2 signalling that has been shown to improve aspects of the skeletal muscle pathology in dystrophic mice. Whether SFN can similarly improve GI function in muscular dystrophy was unknown. Video imaging and spatiotemporal mapping to assess gastrointestinal contractions in isolated colon preparations from *mdx* and C57BL/10 mice revealed that SFN reduced contraction frequency when administered ex vivo, demonstrating its therapeutic potential to improve GI function in DMD. To confirm this in vivo, four-week-old male C57BL/10 and *mdx* mice received vehicle (2% DMSO/corn oil) or SFN (2 mg/kg in 2% DMSO/corn oil) via daily oral gavage five days/week for 4 weeks. SFN administration reduced fibrosis in the diaphragm of *mdx* mice but did not affect other pathological markers. Gene and protein analysis revealed no change in Nrf2 protein expression or activation of Nrf2 signalling after SFN administration and oral SFN supplementation did not improve GI function in *mdx* mice. Although ex vivo studies demonstrate SFN’s therapeutic potential for reducing colon contractions, in vivo studies should investigate higher doses and/or alternate routes of administration to confirm SFN’s potential to improve GI function in DMD.

## 1. Introduction

Duchenne muscular dystrophy (DMD) is associated with clinical manifestations of abnormal gastric and colonic motor activities [1,2,3,4,5,6,7,8]. Constipation, bloating, and feelings of fullness are reported in patients with DMD and other muscular dystrophies, attributed to delayed gastric emptying, gastroesophageal reflux (GER), and constipation [9]. One study reported GI symptoms in 47% of 118 DMD patients aged 3–35 years [10]. These GI issues were generally ascribed to the immobility associated with the progression of DMD, but since dystrophin is also expressed in neural and smooth muscle tissue of the gut [11] and because GI abnormalities have been described in *mdx* dystrophic mice, the most widely used animal model of DMD [12,13,14,15], a lack of dystrophin could also contribute to GI dysfunction. Studies in *mdx* mice show thickening of the muscularis externa of the colon at 10 weeks of age compared to control mice [16], and both fecal pellet mass and production are reduced in *mdx* compared with control mice [17]. GI motility is altered in *mdx* mice, with various studies reporting changes in constriction and contraction frequency [13,17,18].

Since dystrophin deficiency affects neuronal nitric oxide synthase (nNOS) localisation and nitric oxide (NO) production in muscle fibres, and myogenic NOS and endogenous NO production are defective in the colon of *mdx* mice [17], studies have examined the effect of increased NO signalling on GI function. Furthermore, the addition of relaxin, which modulates NO production, counteracts reduced gastric and small intestinal motility in *mdx* mice [19], and exogenous addition of L-arginine (converted to NO by NOS) to isolated colons from *mdx* mice restores normal motor activity [20]. In addition to altered NO signalling, chronic inflammation and associated oxidative stress are key hallmarks of the dystrophic pathology [21]. Production of inflammatory cytokines, such as interleukin-6 (IL-6), modifies the absorpto-secretory function and contractile activity of the colon [22,23], and so factors able to reduce inflammation and associated oxidative stress have therapeutic potential to improve GI function in DMD. Consistent with this hypothesis, neutralising IL-6 signalling improves gut function in *mdx* mice [16].

Sulforaphane (SFN), an isothiocyanate derived from glucoraphanin found in vegetables of the cruciferous family, has powerful chemoprotective [24] and neuroprotective properties largely attributed to its antioxidant effects via regulation of NF-E2 related factor 2 (Nrf2; [25]). Nrf2, in conjunction with Kelch-like-ECH-associated protein (Keap1), acts to regulate antioxidant activity within cells. Under basal conditions, Nrf2 is held in an inactive state through its interaction with Keap1, but in response to oxidative stress, it is released and translocates to the nucleus, binding to the antioxidant response element (ARE) to mediate the transcription of antioxidant enzymes [26]. SFN acts by dissociating Nrf2 from Keap1 to allow Nrf2 to activate the ARE in the nucleus and produce antioxidant enzymes [27]. Importantly, SFN administration and Nrf2 activation have been investigated for ameliorating inflammation and mucosal damage in the GI tract [28,29,30,31,32,33].

Oral SFN administration, at a dose of 2 mg/kg/day, to four week old *mdx* mice improved the dystrophic phenotype [34,35,36] with increased muscle mass, force production, running distance and reduced glutathione/oxidised glutathione (GSH/GSSH) ratio compared to untreated mice [35]. In addition, SFN administration decreased markers of muscle damage, such as creatine kinase (CK), centrally nucleated fibres, inflammation, and fibrosis [35,36]. Importantly, SFN administration had similar effects on *mdx* mice when administered from either three weeks or from 12 weeks of age [34], indicating a larger ‘therapeutic window’ of opportunity for DMD. Whether SFN could similarly reduce inflammation and oxidative stress in the gut to improve colon motility and nutrient uptake had not been investigated.

Using video recording and spatiotemporal (ST) mapping to examine colon motility patterns, we confirmed altered gut function in *mdx* mice and demonstrated that nutritional interventions to increase NO signalling can improve colon motility [37]. Based on previous studies of SFN’s anti-inflammatory effects on skeletal muscles of *mdx* mice [34,35,36], we investigated whether oral administration of SFN could similarly improve GI function in this murine model of DMD.

## 2. Materials and Methods

*Animals*. All experimental protocols were approved by the Animal Ethics Committee of The University of Melbourne and conducted in accordance with the Australian code of practice for the care and use of animals for scientific purposes, as stipulated by the National Health and Medical Research Council (Australia). Experiments were performed on C57Bl/10ScSn (C57BL/10) and C57BL/10ScSn-Dmd*^mdx^*/Arc (*mdx*) four-week-old male mice were obtained from the Animal Resources Centre (ARC Animal Facility, Western Australia). Mice arrived from the ARC at three weeks of age and were housed in boxes of four mice of the same genotype in microisolator cages on a 12 h:12 h light:dark cycle with standard laboratory chow and water available *ad libitum*. All mice received a one week acclimation period prior to the beginning of experimental protocols and all protocols were performed in the light cycle.

*SFN administration*. D,L-Sulforaphane (#574215; Merck Millipore, Darmstadt, Germany) was prepared and administered as per previously published protocols testing SFN administration in *mdx* mice [34,35,36]. SFN was dissolved in DMSO to generate a stock concentration of 10 mg/mL. SFN in DMSO was diluted 1:50 in corn oil to give a concentration of 0.2 mg/mL for delivery via oral gavage. Four-week-old C57BL/10ScSn (*n* = 20) and C57BL/10ScSn *mdx* (*n* = 20) mice received either vehicle (2% DMSO in corn oil; 10 µL/g body mass) or SFN (2 mg/kg in 2% DMSO/corn oil; 10 µL/g body mass) once daily, five days per week for four weeks. Food intake, water intake, and body mass were monitored daily throughout the treatment period. At the conclusion of treatment, one cohort of mice (*n* = 12/group) was killed by cervical dislocation for assessment of colon motility while a second cohort (*n* = 8/group) was dissected under anaesthesia for collection of serum and tissue samples for biochemical analysis.

*Serum enzyme analysis*. Whole blood was collected from mice (*n* = 8/group) into Eppendorf tubes at endpoint via cardiac puncture. To isolate serum, tubes were incubated for 30 min at room temperature, then centrifuged 1500 g for 10 min at 4 °C and serum removed to fresh Eppendorf tubes and stored at −80 °C. Levels of serum CK and aspartate aminotransferase (AST) were measured on a VetScan VS2 chemistry analyser (Abaxis Inc., Union City, CA, USA) using the Equine Profile Plus rotor according to manufacturer’s protocol.

*Analysis of fibrosis*. Serial sections (8 µm) were transversely cut through the costal diaphragm muscle (*n* = 8/group) using a refrigerated (−20 °C) cryostat (CTI Cryostat; IEC, Needham Heights, Needham, MA, USA) and stained with Van Gieson’s to assess collagen infiltration, as described previously [38,39]. Digital images were obtained using an upright microscope with camera (Axio Imager D1; Carl Zeiss AG, Oberkochen, Germany), controlled and quantified using Axio Vision AC software (Axio Vision AC Rel. 4.7.1; Carl Zeiss AG).

*Video recording of colon motility*. C57Bl/10 (*n* = 12) and *mdx* (*n* = 12) mice were killed by cervical dislocation at the conclusion of the treatment period. Colons were excised from caecum to rectum and placed into physiological saline (118 mM NaCl, 4.6 mM KCl, 2.5 mM CaCl_2_, 1.2 mM MgSO_4_, 1 mM NaH_2_PO_4_, 25 mM NaHCO_3_, 11 mM D-glucose) for assessment of colon contractility as described previously [37,40]. Following cannulation, equilibration recordings were taken (2 × 15 min videos), after which the luminal inflow was replaced with fresh physiological saline and contractions recorded at a controlled pressure over the period of 60 min (4 × 15 min videos) using a Logitech Quickcam Pro 9000 camera and the video acquisition software VirtualDub 10.01. After acquisition, *avi* video files were converted into *su2* files using Scribble 2.1 (University of Melbourne, Melbourne, Australia; in-house software), which uses an edge detection algorithm to identify colon diameter for each frame of the 15 min video recording to generate ST maps, used to analyse contraction number and colon diameter at the level of the proximal, mid and distal colon, as described previously [37,40]. 

*Western immunoblotting*. The TA muscles and distal colon tissue (*n* = 4/group) were snap frozen in liquid nitrogen in Eppendorf tubes and stored at −80 °C. Lysis buffer (10 mM Tris-HCl (pH 7.4), 100 mM NaCl, 1 mM EDTA, 1 mM EGTA, 1 mM NaF, 1% Triton, 10% glycerol, 0.1% SDS, 20 mM Na_4_P_2_O_7_, 0.5 mM Na_3_VO_4_, 0.5% sodium deoxycholate, 0.1 mM PMSF and protease and phosphatase inhibitors, all from Sigma-Aldrich, St. Louis, MO, USA) was added at a ratio of 10:1 buffer to tissue weight. Tissues were homogenised in a Precellys 24 high powered bead mill homogeniser (Thermo Fisher Scientific, Waltham, MA, USA) using metal lysis beads (4500 g, 2 × 15 sec bursts, with a 10 sec break). Lysates were centrifuged (10,000 g, 4 °C, 10 min) and protein concentration determined using a DC™ Protein Assay (Bio-Rad, Hercules, CA, USA), with lysates diluted to 2 mg/mL and 4 × Laemmli sample buffer added to each sample. Samples were denatured (95 °C for 3 min) and 20 µg of total protein loaded onto 26-well Bio-Rad Criterion gels (4–15%; Bio-Rad) alongside Precision Plus Protein™ Dual Color Standard (Bio-Rad). Gels were run at 100 V at room temperature and proteins were transferred to polyvinylidene difluoride (PVDF; Immobilon-P, Merck Millipore, Darmstadt, Germany) membranes. Membranes were blocked in 1× Tris Buffered Saline with 0.1% Tween-20 (TBST)/5% Bovine Serum Albumin (BSA, Sigma Aldrich, St. Louis, MO, USA), followed by overnight (o/*n*) incubation at 4 °C in primary antibodies. Membranes were washed in TBST (3 × 5 min), followed by 1 h incubation at RT in secondary antibody and washed in TBST (4 × 10 min). Membranes were developed using SuperSignal™ West Pico Chemiluminescent Substrate (Thermo Fisher Scientific) on a ChemiDoc MP Imaging System (Bio-Rad). Total protein stains were completed using the BLOT-FastStain™ kit according to manufacturer’s instructions (G-Biosciences, St. Louis, MO, USA) and quantification performed using Image Lab 4.1 software (Bio-Rad).

*Antibodies*. The following primary antibodies were used throughout the experiments in TBST/5% BSA: mouse-α-dystrophin MANEX1011B (1C7) (deposited by Morris, G.E; Developmental Studies Hybridoma Bank, The University of Iowa, Iowa City, IA; USA; 1:1000), rabbit-α-Nrf2 (#12721; Cell Signaling Technologies, Danvers, MA, USA), and mouse-α-4,hydroxynoneal [HNEJ-2] (#ab48506; Abcam, Cambridge, MA, USA). Horseradish peroxidase (HRP)-conjugated sheep-α-mouse IgG and donkey-α-rabbit IgG (GE Healthcare Life Sciences; Marlborough, MA, USA) were used at 1:5000 in TBST/5% BSA.

*Real time quantitative PCR*. Total RNA was extracted from the diaphragm muscle (*n* = 6/genotype/timepoint) using TRIzol/chloroform followed by the RNeasy Fibrous Tissue Mini Kit (Qiagen) as per manufacturer’s instructions. The concentration and quality of RNA samples was determined using Nanodrop 2000 spectrophotometer (Thermo Scientific, Waltham, MA, USA). Real-time RT-PCR was performed as described previously [39,41] using the using the forward and reverse primer sequences described in Table 1. Gene expression was quantified using a cycle threshold (C_T_) method. Relative gene expression was calculated using the expression 2^−ΔCT^, normalized to total cDNA content as determined using Qubit 2.0 Fluorometer (Life Technologies, Carlsbad, CA, USA), as described previously [42].

*Statistics*. Differences between genotype and treatment were assessed using a two-way ANOVA with Bonferroni’s post-hoc multiple comparisons test. Differences in contraction number between two groups were assessed using a Mann Whitney U test or a Wilcoxon matched-pairs signed rank test where appropriate. Differences in contraction number between multiple treatment groups were assessed using a Kruskal-Wallis test. A *p* value less than 0.05 was considered statistically significant. All statistical analyses were carried out using GraphPad Prism 6 software (GraphPad Software Inc., La Jolla, CA, USA). All values are mean ± standard error of mean (SEM).

## 3. Results

### 3.1. Sulforaphane Reduces Number of Contractions in Excised Colon Preparations Ex Vivo

**Figure 1:**
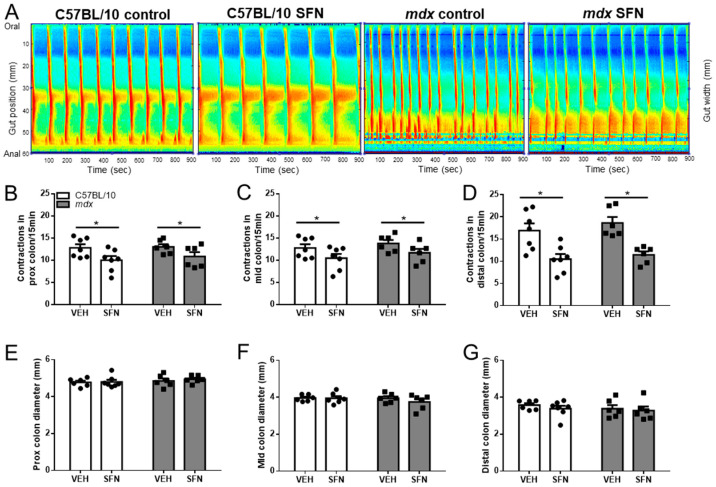
Ex vivo SFN treatment reduces contraction number in the distal colon. (**A**) Colons were excised from eight-week-old male C57BL/10 and *mdx* mice to assess ex vivo contraction by video recording and ST mapping of colon diameter during a control period (4 × 15 min; VEH) and subsequently in the presence of 5 µM SFN (4 × 15 min; SFN). Contraction frequency (number of contractions per 15 min recording) was determined for the proximal (**B**), mid (**C**), and distal colon (**D**). Statistical analysis was performed using a Wilcoxon matched-pairs signed rank test to assess effects of treatment within each genotype. Resting diameter of the proximal (**E**), mid (**F**), and distal (**G**) colon was measured from the ST maps. Statistical analysis was performed using a two-way ANOVA with a Bonferroni’s post-hoc test to assess effects of treatment. *n* = 6–7/genotype/group. * *p* < 0.05.

To determine the potential for SFN to improve GI function in dystrophic mice, colons were excised from C57BL/10 and *mdx* mice and contractility of the proximal, mid, and distal colon was assessed in an organ bath ex vivo in the presence of physiological saline (control) and subsequently in physiological saline with 5 µM SFN. Although we have previously reported increased contraction number in the distal colon of *mdx* mice [37], the number of mice used for this study was not sufficient to demonstrate a significant difference in contraction number between C57BL/10 and *mdx* mice in the control period (Figure 1A–D). However, addition of SFN to the bathing medium reduced contraction number in the proximal (Figure 1A,B), mid (Figure 1A,C) and distal regions of isolated colons (Figure 1A,D) from both C57BL/10 and *mdx* mice compared to the control period (in saline only). SFN did not alter the resting diameter of the proximal (Figure 1A,E), mid (Figure 1A,F) or distal (Figure 1A,G) colon. The reduction in contraction number after addition of SFN ex vivo, indicated therapeutic potential to reduce abnormal contractility in the distal colon of *mdx* mice and warranted further examination in vivo.

### 3.2. Oral Sulforaphane Supplementation Does Not Ameliorate Gastrointestinal Dysfunction in mdx Mice

**Figure 2:**
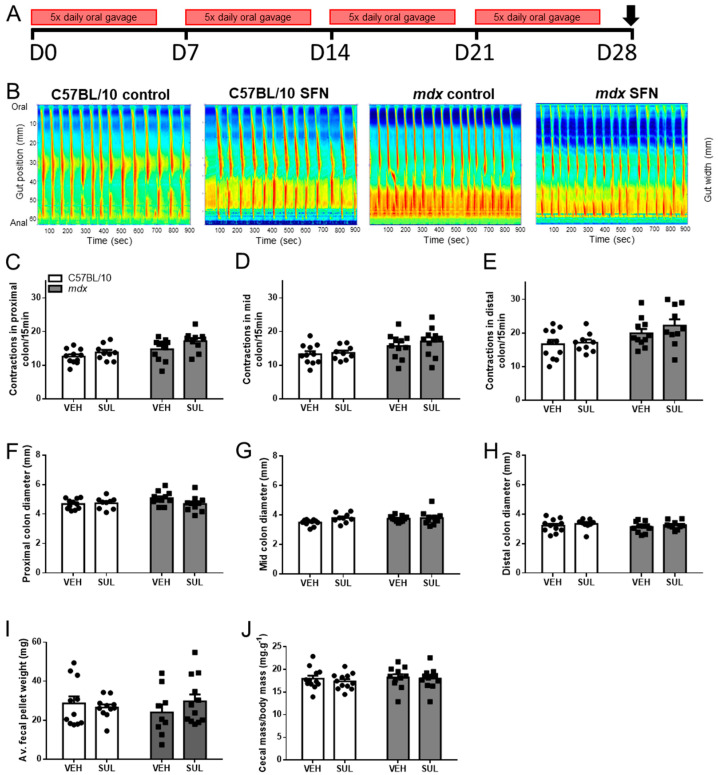
Oral SFN administration does not improve gastrointestinal function in *mdx* mice. (**A**) Four-week-old male C57BL/10 and *mdx* mice received either vehicle (2% DMSO/corn oil; VEH) or SFN (2 mg/kg in 2% DMSO/corn oil; SFN) via daily oral gavage for five days a week for four weeks. (**B**) Contraction frequency (number of contractions per 15 min recording) was determined for the proximal (**C**), mid (**D**), and distal colon (**E**). Statistical analysis was performed using a Kruskal-Wallis test to assess effects of treatment. *n* = 9–12/genotype/group. Resting diameter of the proximal (**F**), mid (**G**), and distal (**H**) colon was measured from the ST maps. Fecal pellet wet weight (**I**), and caecum mass (**J**). Statistical analysis was performed using a two-way ANOVA with a Bonferroni’s post-hoc test to assess effects of treatment. *n* = 9–12/genotype/group.

We have reported previously that *mdx* mice exhibit gastrointestinal dysfunction from four weeks of age [37], as evidence by an increased number of contractions in the distal colon. To determine whether oral SFN supplementation could attenuate GI dysfunction in *mdx* mice, four-week-old C57BL/10 and *mdx* mice received either corn oil (vehicle) or 2 mg/kg SFN via daily oral gavage five times/week for four weeks (Figure 2A), and the number of contractions in proximal, mid and distal segments of isolated colon preparations was evaluated using video imaging and ST mapping. No difference was observed in the number of contractions in the proximal (Figure 2B,C), mid (Figure 2B,D), or distal (Figure 2B,E) colons of *mdx* mice relative to C57BL/10 receiving either vehicle or SFN. No changes were observed in the resting diameter of the proximal (Figure 2F), mid (Figure 2G), or distal (Figure 2H) colon in *mdx* mice compared with C57BL/10 mice. No change was detected in fecal pellet mass (Figure 2I) or cecal mass (Figure 2J). No difference in colon contraction number was observed between vehicle treated C57BL/10 and *mdx* mice (Figure 2B–E). As contraction number has been reported to be higher in the distal colon of *mdx* mice compared to C57BL10 mice [37], we next assessed whether administration of the corn oil vehicle was responsible for increasing contraction number in C57BL/10 mice. 4-week-old C57BL/10 mice received either nothing (control) or 2% DMSO in corn oil via 5× daily oral gavage for four weeks (Supplementary Material Figure S1A) and contraction number in the proximal, mid and distal colon segments was assessed. Administration of corn oil increased contraction number in the mid and distal colon (Figure S1B,C), but had no impact on resting colon diameter (Figure S1B,D).

### 3.3. Oral Sulforaphane Supplementation Reduces Diaphragm Fibrosis but Not Other Markers of Dystrophic Pathology in mdx Mice

**Figure 3:**
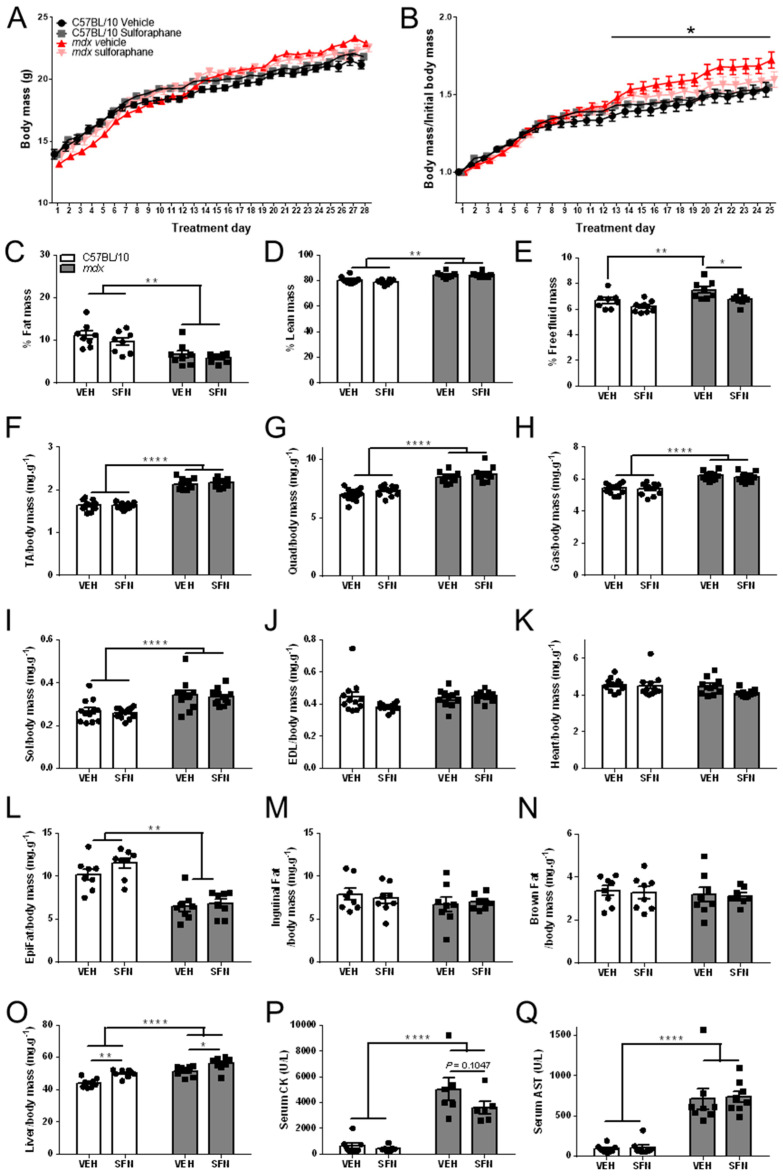
Oral SFN administration does not alter muscle or organ mass in *mdx* mice. Four-week-old male C57BL/10 and *mdx* mice received either vehicle (2% DMSO/corn oil; VEH) or SFN (2 mg/kg in 2% DMSO/corn oil; SFN) via daily oral gavage for five days a week for four weeks. Body mass (**A**) was measured daily over the treatment period and assessed relative to starting body mass (**B**). Statistical analysis was performed using a repeated measures two-way ANOVA with a Bonferroni’s post-hoc test to assess the effects of treatment. *n* = 20/group. Body composition was analysed prior to dissection to measure the % fat mass (**C**), % lean mass (**D**) and % free fluid mass (**E**) of all mice. Statistical analysis was performed using a two-way ANOVA with a Bonferroni’s post-hoc test to assess effects of treatment. *n* = 8/group. Mass of the tibialis anterior (TA; (**F**)), quadriceps (Quad; (**G**)), gastrocnemius (Gas; (**H**)), soleus (sol; (**I**)), extensor digitorum longus (EDL; (**J**)) muscles as well as the heart (**K**), epididymal fat (**L**), inguinal fat (**M**), brown fat (**N**), and liver (**O**) masses relative to body mass were determined at endpoint. *n* = 10–12/group. Serum levels of CK (**P**) and AST (**Q**). *n* = 6–8/group. * *p* < 0.05, ** *p* < 0.01, **** *p* < 0.0001.

As 2 mg/kg/day SFN was shown previously to improve dystrophic pathology in *mdx* mice [34,35,36], we sought to confirm this finding. Body mass of all mice increased each day over the 4-week treatment period (Figure 3A). When normalised to starting body mass, vehicle-treated *mdx* mice gained more weight than C57BL/10 mice and this was normalised in SFN-treated *mdx* mice (Figure 3B). This was not due to alterations in food intake as while SFN showed a trend towards decreasing food intake in C57BL/10 mice (*p* = 0.1087), it had no impact on food intake in *mdx* mice (Figure S2). To determine whether this was due to changes in fat or lean mass, body composition was analysed at the conclusion of treatment. 

As a percentage of total body mass, fat mass (Figure 3C) was reduced and lean mass (Figure 3D) was increased in *mdx* mice relative to C57BL/10 mice. Neither parameter was altered by SFN administration at this dose (Figure 3C,D). In contrast, free fluid mass, which constitutes the remainder of body mass, was increased in vehicle treated *mdx* mice relative to C57BL/10 mice and was reduced by SFN administration (Figure 3E).

**Figure 4:**
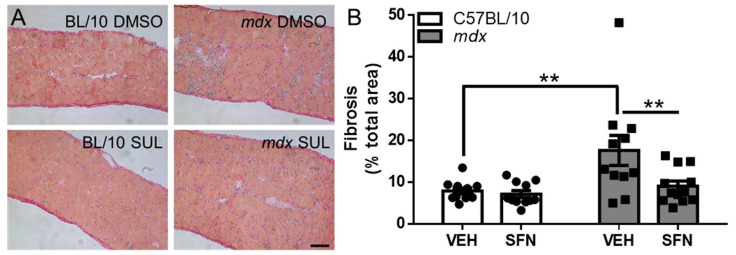
Oral SFN administration reduces fibrosis in the diaphragm of *mdx* mice. Four-week-old male C57BL/10 and *mdx* mice received either vehicle (2% DMSO/corn oil; VEH) or SFN (2 mg/kg in 2% DMSO/corn oil; SFN) via daily oral gavage for five days a week for four weeks. Diaphragm muscle fibrosis (**A**) was quantified (**B**) at the conclusion of treatment. *n* = 11–12/group. Scale bar = 100 µM. Statistical analysis was performed using a two-way ANOVA with a Bonferroni’s post-hoc test to assess effects of treatment. ** *p* < 0.01.

At the conclusion of the four-week treatment period, mice were killed by cardiac excision under anaesthetic and the skeletal muscles and organs were excised and weighed. Relative to body mass, the mass of the tibialis anterior (TA; Figure 3F), quadriceps (Quad; Figure 3G), gastrocnemius (Gas; Figure 3H), and soleus (Sol; Figure 3I) muscles was greater in *mdx* mice relative to C57BL/10 mice, in line with the known pathological features of the *mdx* mouse, and this was not altered with SFN administration. Mass of the extensor digitorum longus (EDL; Figure 3J) muscles and the heart (Figure 3K) were not changed in *mdx* mice relative to C57BL/10 mice or altered with SFN administration. Epididymal fat (Figure 3L), but not inguinal fat (Figure 3M) or brown fat (Figure 3N), was reduced in *mdx* mice relative to C57BL/10 mice and was not altered by SFN administration. However, liver mass relative to body mass was larger in *mdx* mice relative to C57BL/10 mice and increased by SFN administration in both genotypes (Figure 3O). Serum levels of CK (Figure 3P) and AST (Figure 3Q), both known to be elevated in DMD, were higher in *mdx* mice relative to C57BL/10 mice and not reduced with SFN administration, although there was a trend towards reduction (*p* = 0.1047). Fibrosis in the diaphragm was increased in vehicle-treated *mdx* mice compared with control mice and reduced by SFN administration (Figure 4A,B). Together, these data demonstrate that SFN administration (2 mg/kg/day) from four weeks to eight weeks of age, alleviated diaphragm fibrosis but had no effect on any other pathological markers in *mdx* mice.

### 3.4. Oral Sulforaphane Supplementation Did Not Activate Nrf2 Signalling in C57BL/10 or mdx Mice

**Figure 5:**
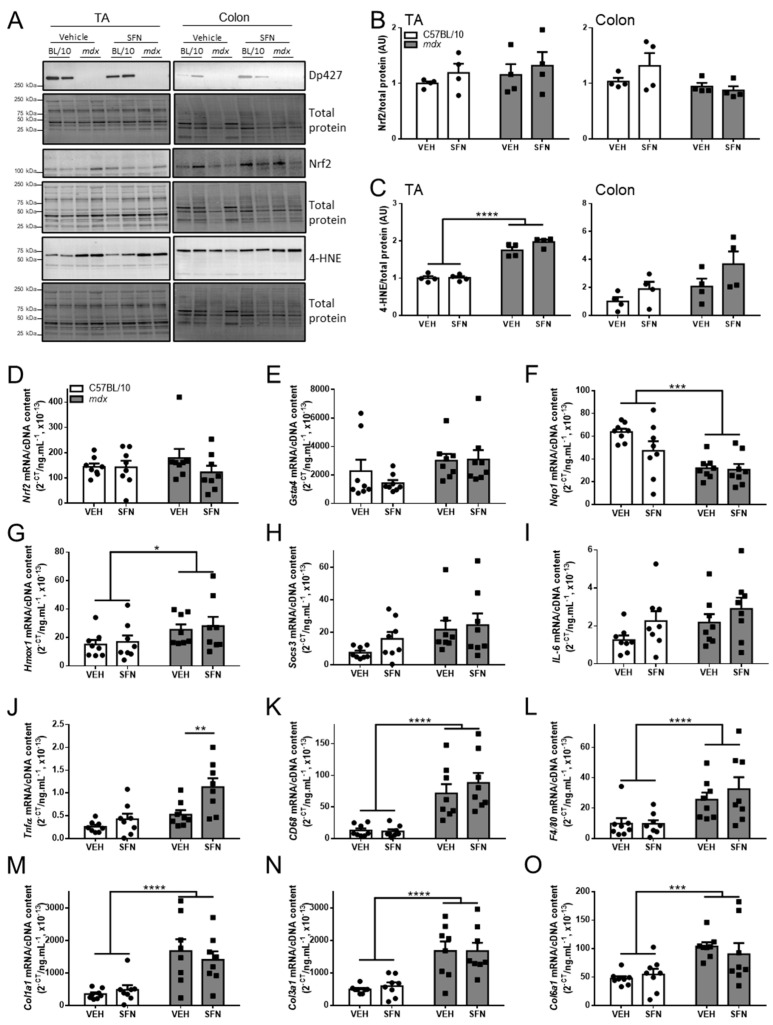
Oral SFN administration does not activate Nrf2 signalling or reduce oxidative stress in limb muscles or the colon. Four-week-old male C57BL/10 and *mdx* mice received either vehicle (2% DMSO/corn oil; VEH) or SFN (2 mg/kg in 2% DMSO/corn oil; SFN) via daily oral gavage for five days a week for four weeks. Expression of dystrophin Dp427, Nrf2, and 4-HNE (**A**). Levels of Nrf2 protein (**B**) and 4-HNE (**C**) were quantified and expressed relative to levels of total protein. *N* = 4/group. RNA was extracted from the diaphragm and RT-PCR used to examine gene expression of *Nrf2* (**D**), *Gsta4* (**E**), *Nqo1* (**F**), *Hmox1* (**G**), *Socs3* (**H**), *IL-6* (**I**), *Tnf-α* (**J**), *CD68* (**K**), *F4/80* (**L**), *Col1a1* (**M**), *Col3a1* (**N**), and *Col6a1* (**O**). *n* = 8/group. Statistical analysis was performed using a two-way ANOVA with a Bonferroni’s post-hoc test to assess effects of treatment. * *p* < 0.05, ** *p* < 0.01, *** *p* < 0.001, **** *p* < 0.0001.

To determine why SFN did not improve GI function in *mdx* mice after oral supplementation, we examined the expression of factors known to be increased by SFN or elevated in *mdx* mice, in limb muscle and the distal colon. Western immunoblotting confirmed expression of the dystrophin Dp427 isoform in the TA muscle and colon of C57BL/10 mice and absence in *mdx* mice (Figure 5A). Protein expression of Nrf2 was confirmed in the TA muscle and colon of both C57BL/10 and *mdx* mice but this was not increased after SFN administration (Figure 5A,B). Levels of 4-HNE modified proteins, an indicator of oxidative stress, were increased in the TA muscle but not the colon of *mdx* mice relative to controls and were not changed with SFN administration (Figure 5A,C). Together, these findings indicate that SFN did not activate the Nrf2 signalling pathway in the limb muscles or colon of either C57BL/10 or *mdx* mice. As the diaphragm was the only muscle to demonstrate an improvement in *mdx* mice after oral SFN administration at 2 mg/kg/day, we next assessed gene expression of markers of the Nrf2 signalling pathway, inflammation, and fibrosis. Neither gene expression of *Nrf2* (Figure 5D), *Gsta4* (Figure 5E), *Nqo1* (Figure 5F), nor *Hmox1* (Figure 5G) were altered following SFN administration, but *Nqo1* (Figure 5F) was decreased and *Hmox1* (Figure 5G) increased in *mdx* mice relative to C57BL/10 mice. Gene expression of the inflammatory markers *Socs3* (Figure 5H) and *IL-6* (Figure 5I) were not altered in *mdx* mice relative to C57BL/10 mice and not changed after SFN administration. *Tnfα* gene expression was not changed in *mdx* mice but was increased by SFN (Figure 5J). Gene expression of both *CD68* (Figure 5K) and *F4/80* (Figure 5L) were increased in *mdx* mice relative to C57BL/10 mice but were not altered by SFN. Lastly, as fibrosis was decreased in the diaphragm of SFN treated *mdx* mice, we assessed gene expression of collagen markers and found *Col1a1* (Figure 5M), *Col3a1* (Figure 5N), and *Col6a1* (Figure 5O) were elevated in *mdx* mice but were not reduced with SFN. These data demonstrate that oral SFN supplementation at a dose of 2 mg/kg/day did not activate Nrf2 signalling nor did it reduce gene expression of markers of dystrophic pathology in either the colon or the skeletal muscle.

The lack of response to SFN in the mice was investigated further in experiments performed on C2C12 myotubes treated with SFN at doses of 5 µM and 10 µM. As shown previously, LPS increased *IL-6*, *Socs3*, and *TNFα* gene expression in C2C12 myotubes, which were all decreased with administration of 10 µM SFN (Figure S3A–C). SFN did not increase *Nrf2* gene expression (Figure S3D) but did increase expression of the Nrf2 target genes *Nqo1* (Figure S3E) and *Hmox1* (Figure S3F) at both 5 µM and 10 µM SFN, in the presence and absence of LPS, indicating activation of Nrf2 signalling. In addition, Nrf2 protein expression was increased after 1 h incubation with 5 µM and 10 µM SFN in healthy C2C12 and dystrophin-deficient C2C12 myotubes in vitro (Figure S3G). Together, these data confirm that SFN increased Nrf2 protein expression and activated Nrf2-mediated antioxidant signalling in vitro.

## 4. Discussion

GI dysfunction is a co-morbidity in DMD patients and similarly affects the *mdx* dystrophic mouse. We and others have reported that reducing inflammation or restoring nNOS signalling can improve GI function in *mdx* mice [16,17,19,20,37]. SFN, administered at a dose of 2 mg/kg/day via oral gavage, had been shown by others to improve multiple aspects of dystrophic pathology in the *mdx* mouse [34,35,36]. As SFN is a known antioxidant with anti-inflammatory properties, we sought to determine whether this treatment could restore GI function in dystrophic mice. Administration of 5 µM SFN reduced contraction numbers across the length of the colon in colons excised from both C57BL/10 and *mdx* mice when administered ex vivo, suggesting that SFN has the potential to reduce colon contraction frequency even in the absence of a dystrophic phenotype. However, while oral administration with 2 mg/kg/day SFN for 4 weeks reduced diaphragm fibrosis, it did not alter any other parameters of the dystrophic pathology and did not improve colon contractility in *mdx* mice. Therefore, although SFN demonstrates therapeutic potential to improve GI function in DMD, the optimal dose and route of administration required to confer this effect in *mdx* mice in vivo, remains to be determined.

Oral supplementation with SFN, at a dose of 2 mg/kg/day, was shown previously by others to improve multiple aspects of dystrophic pathology in *mdx* mice [34,35,36]. These studies utilised three different periods of treatment, demonstrating improvements in dystrophic parameters in four-week-old mice after an eight-week treatment period [35], and improvements in 12-week-old mice after a three-month treatment period [34]. In the present study, mice were treated for four-weeks and unsurprisingly the improvements in dystrophic pathology were less than what has been reported. However, four-weeks of SFN administration to four-week-old mice had been shown to activate anti-oxidant signalling via Nrf2 [36], indicating that the duration of treatment we employed should have been sufficient to activate signalling and improve GI function. We confirmed in C2C12 myotubes that SFN reduced markers of inflammatory signalling, increased markers of Nrf2 signalling, and increased Nrf2 protein expression in a dose-dependent manner, therefore confirming that our SFN stock was biologically active. The lack of an effect of oral SFN administration on GI function in *mdx* mice could be attributed to an inability of SFN to access colonic cells after oral administration or that the dose used was insufficient to elicit an effect. It is also worth noting that SFN administration increased liver mass in both C57BL/10 and *mdx* mice in this study, suggesting an impact of SFN in the liver. While this was not explored in the current study, it warrants further investigation. 

Despite improvements in diaphragm fibrosis in treated *mdx* mice, which were in line with previous reports [34,35,36], we were unable to detect neither increased Nrf2 protein expression nor evidence of Nrf2 signalling after SFN administration in the skeletal muscle or colon. Tissue-specific analyses of SFN uptake after oral administration in rats revealed low uptake in the colon and several other tissues, relative to the stomach where uptake was greatest [43]. Together with the high bioavailability and rapid clearance of SFN from the plasma [44,45,46], this suggests that the dose used in the present study was insufficient to activate Nrf2 signalling in *mdx* mice. Importantly, using the Reagan-Shaw equation [47], the dose used in this study (which equates to 11.28 µmol/kg) translates to a human equivalent dose (HED) of 0.24 mg/kg/day (1.35 µmol/kg). Since broccoli sprouts are reported to contain as much as 1150 mg SFN per 100 g sprouts, this potentially falls short of the dose that could be achieved in the diet. However, it is worth noting that, while on the lower end, this dose is within the dose range previously reported to be effective following oral administration in mice and within the tolerable range in humans [48].

The ability to address whether SFN could improve GI function in *mdx* mice was limited by the inability to demonstrate increased colon contraction frequency in vehicle-treated *mdx* mice, because corn oil used was used as the delivery vehicle for SFN increased contraction frequency in colons from C57BL/10 mice. However, it is important to note that SFN failed to reduce colon contraction number in both C57BL/10 and *mdx* mice after in vivo administration. Despite the lack of effect in the in vivo studies, our ex vivo experiments showing addition of SFN could reduce the contraction number in isolated colons, highlight SFN’s therapeutic potential to improve GI function in DMD, if a suitable dose and route of administration can be identified. 

Based on the findings that oral SFN administration reduces non-steroidal anti-inflammatory drugs (NSAIDs) induced oxidative stress in the small intestine [29,33], and suppress gastrointestinal polyp formation in Apc^min^ mice [49], SFN has potential to improve GI health. It has previously been well established that the response to various neurotransmitters is altered in the *mdx* colon relative to control mice. As dystrophin has a key function in the localisation of nNOS, it has been extensively demonstrated by both ourselves and others that the response to the inhibitory neurotransmitter, NO, is altered in the *mdx* colon and that this is responsible for the majority of the contractile phenotype in the *mdx* colon as restoration of this signalling pathway (via administration of NO donors) restores normal contractile function [13,14,17,19,20,37]. More recently, one group reported reduced contractile response to acetylcholine in isolated colon strips and colonic smooth muscle cells from *mdx* mice [50]. This is perhaps not surprising since dystrophin and its associated complex has been reported to be essential for maturation and clustering of acetylcholine receptors (AChR), albeit at the neuromuscular junction (reviewed in [51]). The potential for SFN to restore the response of the colon to these neurotransmitters in *mdx* mice remains to be determined.

## 5. Conclusions

Other studies have demonstrated improvements in muscle pathologies after in vivo administration of SFN to mice. At a dose of 0.5 mg/kg via daily intraperitoneal injection for one month, SFN treatment increased skeletal muscle mass and protected against loss of muscle mass and strength in diabetic *db*/*db* mice [52]. In a separate study, SFN administered as a supplement in the food at a dose of 442.5 mg/kg body weight for 12 weeks improved exercise capacity and reduced markers of skeletal muscle ageing (sarcopenia), oxidation, and apoptosis in 21–22 month old mice [53]. In both studies, increased Nrf2 signalling in skeletal muscle was confirmed after SFN administration. Together, these data, in conjunction with earlier studies examining skeletal muscle health [34,35,36,54,55], demonstrate that increasing the dose and potentially altering the route of administration may enable SFN to reach the colon and improve motility in *mdx* mice in vivo. Although we showed that SFN has the potential to reduce colon contractility in *mdx* mice, oral SFN supplementation at a dose of 2 mg/kg/day had no positive effect on gastrointestinal physiology in *mdx* mice. Additional in vivo studies are required to optimise the dose and route of administration to activate Nrf2 signalling sufficiently to confer therapeutic benefits for GI dysfunction in DMD.

## Figures and Tables

**Table 1:** Primer sequences used for PCR analysis.

Gene	Forward Primer (5′-3′)	Reverse Primer (5′-3′)
*Socs3*	GCTGGCCAAAGAAATAACCA	AGCTCACCAGCCTCATCTGT-3
*IL-6*	CCGGAGAGGAGACTTCACAG	TCCACGATTTCCCAGAGAAC
*TNFα*	GGCCTTCCTACCTTCAGACC	AGCAAAAGAGGAGGCAACAA
*CD68*	TCCAAGCCCAAATTCAAATC	ATTGTATTCCACCGCCATGT
*F4/80*	CATCAGCCATGTGGGTACAG	CATCACTGCCTCCACTAGCA
*Nfe2l2*	CGCTGGAAAAAGAAGTGGGC	AGTGACTGACTGATGGCAGC
*Nqo1*	AGCCAATCAGCGTTCGGTAT	GCCTCCTTCATGGCGTAGTT
*Hmox1*	GAACCCAGTCTATGCCCCAC	GCGTGCAAGGGATGATTTCC
*Col1a1*	CACCCTCAAGAGCCTGAGTC	GTTCGGGCTGATGTACCAGT
*Col3a1*	ACCAAAAGGTGATGCTGGAC	GACCTCGTGCTCCAGTTAGC
*Col6a1*	CCCCATTGGACCTAAAGGAT	TCTCCCACTTCACCCTCATC

## Data Availability

All relevant data are within the manuscript and its Supporting Information files.

## Supplementary Materials

The following are available online at https://www.mdpi.com/article/10.3390/nu13124559/s1, Materials and methods, Figure S1: Corn oil increases contraction frequency in isolated colons from C57BL/10 mice, Figure S2: SFN does not alter food intake in C57BL/10 or mdx mice, Figure S3: SFN activates Nrf2 signalling and increases Nrf2 protein expression in C2C12 mouse muscle myotubes in vitro.
