# Supplementary material for: Investigating the Potential for Sulforaphane to Attenuate Gastrointestinal Dysfunction in mdx Dystrophic Mice
Source: Nutrients. 2021 Dec 20;13(12):4559. doi: 10.3390/nu13124559 (PMC8706299; doi:10.3390/nu13124559)
Supplement: Supplementary file 1 [file nutrients-13-04559-s001.zip › nutrients-1480536-supplementary.pdf]

Investigating the potential for sulforaphane to attenuate gastrointestinal dysfunction in *mdx* dystrophic mice

Kristy Swiderski, Suzannah J. Read, Audrey Chan, Jin D. Chung, Jennifer Trieu, Timur Naim, Rene Koopman, Gordon S. Lynch*

Supplementary information

Supplementary methods

C2C12 cell culture

The C2C12 myoblast cell line (ATCC, Manassas, VA, USA) was maintained in DMEM supplemented with 10% FBS and 1% L-glutamine on tissue culture plates at 37°C + 5% CO_2 and sub-cultured upon reaching approximately 50-60% confluency. For differentiation into myotubes, C2C12 myoblasts were grown to confluency then incubated in differentiation media (DMEM supplemented with 1% L-glutamine and 2% horse serum (HS; Life Technologies, Scoresby, VIC, Australia) for 4 days at 37°C + 5% CO2, with media refreshed every 48 h. On day 4 differentiation, cells were exposed to differentiation media (DM; control), or DM supplemented with 1 µg/mL lipopolysaccharide (LPS; #L2654, Sigma-Aldrich, St. Louis, MO, USA) in the presence or absence of 5 µM or 10 µM SFN for 2h.

Protein extraction from C2C12 myotubes

C2C12 myoblasts were grown to confluency then incubated in differentiation media (DMEM supplemented with 1% L-glutamine and 2% horse serum (HS; Life Technologies, Scoresby, VIC, Australia) for 4 days at 37°C + 5% CO2, with media refreshed every 48 h. On day 4 differentiation, cells were exposed to differentiation media (DM; control), or 5 µM or 10 µM SFN for 1h. Cells were lysed in RIPA lysis buffer (#20-188; Merck Millipore, Darmstadt, Germany) supplemented with protease inhibitor cocktail (PIC; #P2714-1BTL; Sigma-Aldrich) on ice, sonicated 1× 15 sec pulse on setting 7 using a Microson ultrasonic cell disruptor XL2000 (Misonix, New York, USA), and centrifuged 10000 rpm 10 min 4°C. Lysate was diluted in 4× Laemmli sample buffer and heated 95°C 3 min before being subjected to SDS-PAGE and western immunoblotting.

Supplementary figure legends

Figure S1. Corn oil increases contraction frequency in isolated colons from C57BL/10 mice. (A) 4-week-old male C57BL/10 mice were left untreated or received 2% DMSO/corn oil via daily oral gavage for 5 days a week for 4 weeks. (B) After treatment, colons were excised and contractions assessed *ex vivo* by video recording and ST mapping of colon diameter. (C) Contraction frequency (number of contractions per 15 min recording) was determined for the proximal, mid, and the distal colon. Statistical analysis was performed using a Mann Whitney U test to assess effects of treatment. n=8/group. $^*P < 0.05$, $^{**}P < 0.01$. (D) Resting diameter of the proximal, mid, and distal colon was measured from the ST maps. Statistical analysis was performed using a two-way ANOVA with a Bonferroni's post-hoc test to assess effects of treatment. n=8/group.

Figure S2. SFN does not alter food intake in C57BL/10 or *mdx* mice. 4-week-old male C57BL/10 mice were left untreated or received 2% DMSO/corn oil via daily oral gavage for 5 days a week for 4 weeks. The amount of food consumed per cage was determined each day and averaged to determine average food intake over the treatment period for each mouse. Statistical analysis was performed using a two-way ANOVA with a Bonferroni's post-hoc test to assess effects of treatment. n=6 cages/group.

Figure S3. SFN activates Nrf2 signaling and increases Nrf2 protein expression in C2C12 mouse muscle myotubes *in vitro*. C2C12 myotubes were left untreated or exposed to 1 µg/mL LPS in the presence or absence of 5 µM or 10 µM SFN for 2 h. Gene expression of *IL-6* (A), *Socs3* (B), *Tnf-α* (C), *Nrf2* (D), *Nqo1* (E), and *Hmox1* (F) was measured relative to total cDNA content. (G) C2C12 myotubes were left untreated or exposed to 5 µM or 10 µM SFN for 1 h, protein was extracted and subjected to SDS-PAGE and western immunoblotting

for Nrf2 protein relative to total protein. Statistical analysis was performed using a two-way ANOVA with a Bonferroni's post-hoc test to assess effects of treatment. *$P < 0.05$, **$P < 0.01$, ***$P < 0.001$, ****$P < 0.0001$ relative to control. n=3/group.

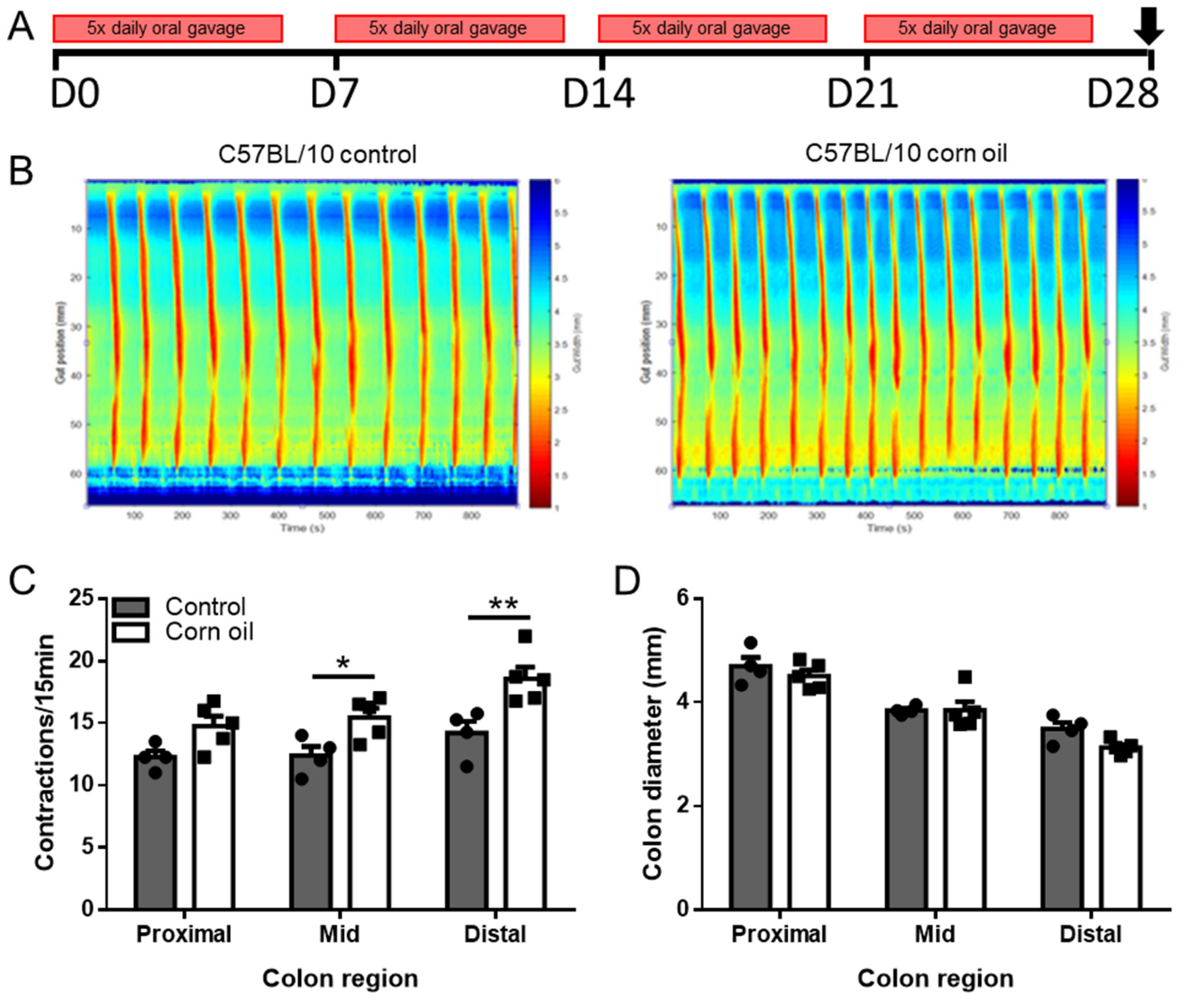

Figure S1

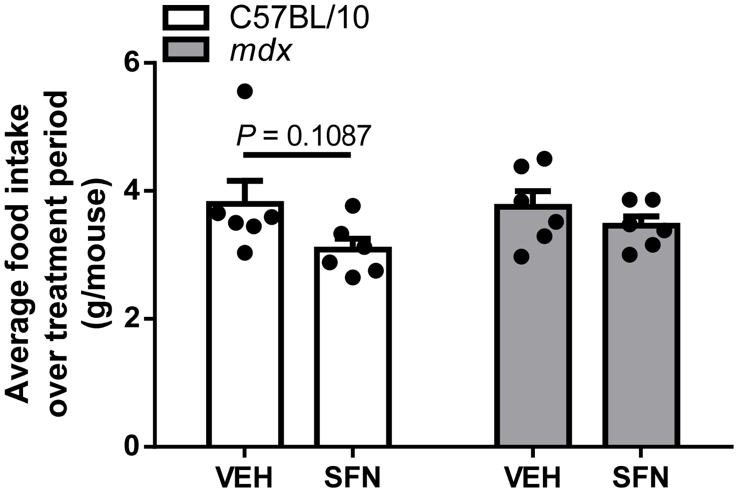
C57BL/10
mdx
Average food intake over treatment period (g/mouse)
P = 0.1087
6
4
2
0
VEH
SFN
VEH
SFN

Figure S2

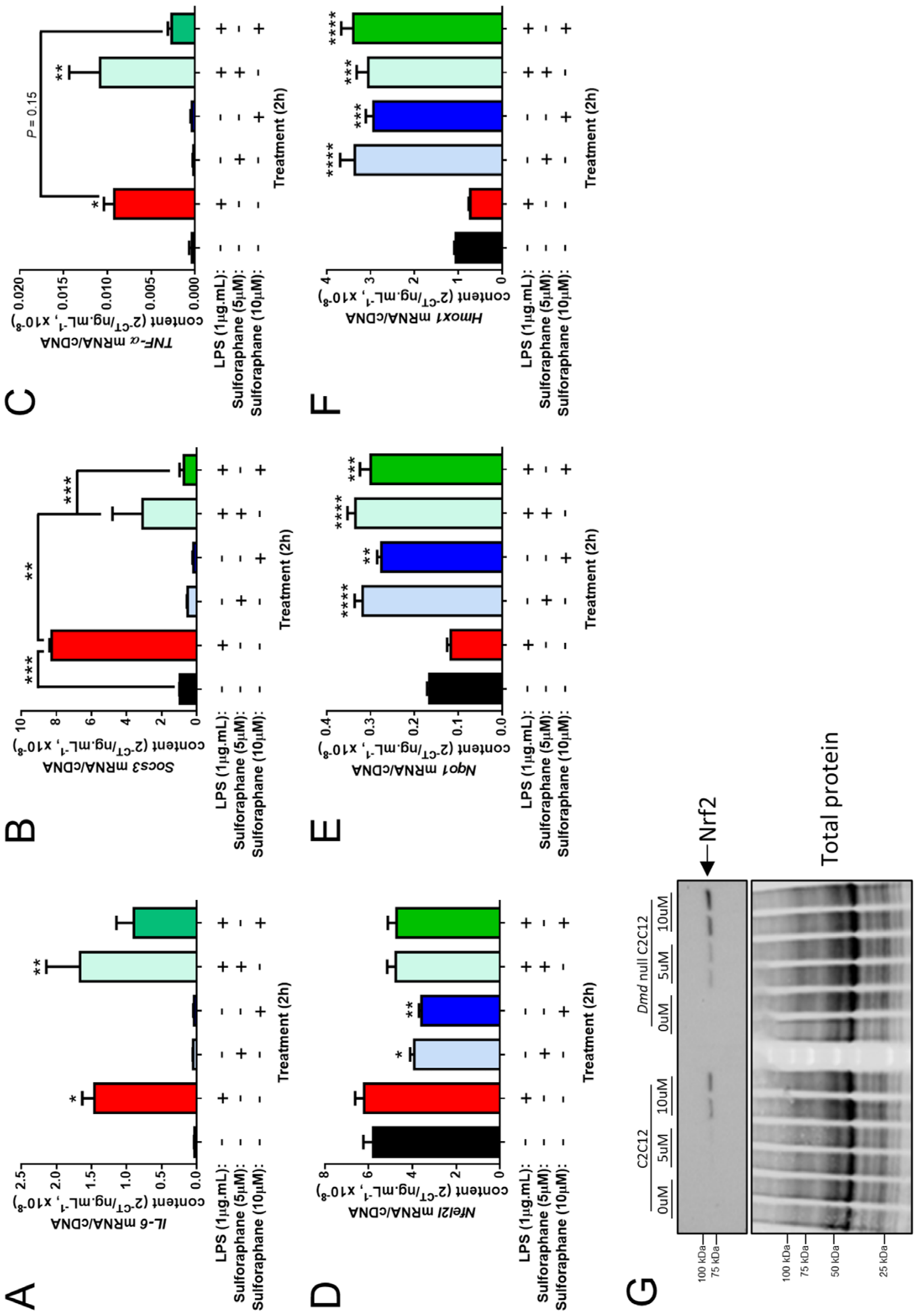
A
IL-6 mRNA/cDNA content (2-CT/ng.mL-1, x10-8)
B
Socs3 mRNA/cDNA content (2-CT/ng.mL-1, x10-8)
C
TNF-α mRNA/cDNA content (2-CT/ng.mL-1, x10-8)
P = 0.15
D
Nfel2l mRNA/cDNA content (2-CT/ng.mL-1, x10-8)
E
Nqo1 mRNA/cDNA content (2-CT/ng.mL-1, x10-8)
F
Hmox1 mRNA/cDNA content (2-CT/ng.mL-1, x10-8)
LPS (1μg.mL):
Sulforaphane (5μM):
Sulforaphane (10μM):
Treatment (2h)
G
C2C12
Dmd null C2C12
0uM
5uM
10uM
Nrf2
Total protein
100 kDa
75 kDa
50 kDa
25 kDa

Figure S3
